# Inter-Tumor Heterogeneity—Melanomas Respond Differently to GM-CSF-Mediated Activation

**DOI:** 10.3390/cells9071683

**Published:** 2020-07-13

**Authors:** Adi Moshe, Sivan Izraely, Orit Sagi-Assif, Sapir Malka, Shlomit Ben-Menachem, Tsipi Meshel, Metsada Pasmanik-Chor, Dave S.B. Hoon, Isaac P. Witz

**Affiliations:** 1 School of Molecular Cell Biology and Biotechnology, George S. Wise Faculty of Life Science, Tel Aviv University, Tel Aviv 6997801, Israel; adi_moshe@msn.com (A.M.); si0881@gmail.com (S.I.); oritassif@gmail.com (O.S.-A.); sapirmalka@mail.tau.ac.il (S.M.); shlulitii@yahoo.com (S.B.-M.); tzipi@post.tau.ac.il (T.M.); 2 Department of Immunology, Weizmann Institute of Science, Rehovot 7610001, Israel; 3 Bioinformatics Unit, The George S. Wise Faculty of Life Science, Tel Aviv University, Tel-Aviv 6997801, Israel; metsada@tauex.tau.ac.il; 4 Department of Translational Molecular Medicine, John Wayne Cancer Institute, Saint John’s Health Center Providence Health Systems, Santa Monica, CA 90404, USA; hoon@jwci.org

**Keywords:** GM-CSF, melanoma, brain metastasis, metastatic microenvironment

## Abstract

Granulocyte-monocyte colony stimulating factor (GM-CSF) is used as an adjuvant in various clinical and preclinical studies with contradictory results. These were attributed to opposing effects of GM-CSF on the immune or myeloid systems of the treated patients or to lack of optimal dosing regimens. The results of the present study point to inter-tumor heterogeneity as a possible mechanism accounting for the contrasting responses to GM-CSF incorporating therapies. Employing xenograft models of human melanomas in nude mice developed in our lab, we detected differential functional responses of melanomas from different patients to GM-CSF both in vitro as well as in vivo. Whereas cells of one melanoma acquired pro metastatic features following exposure to GM-CSF, cells from another melanoma either did not respond or became less malignant. We propose that inter-melanoma heterogeneity as manifested by differential responses of melanoma cells (and perhaps also of other tumor) to GM-CSF may be developed into a predictive marker providing a tool to segregate melanoma patients who will benefit from GM-CSF therapy from those who will not.

## 1. Introduction

Brain metastasis frequently appears in patients with lung and breast cancer as well as in melanoma patients. Due to limited and usually non-effective treatment options, brain metastasis is associated with poor survival and therefore constitutes an unmet clinical challenge [1,2]. In melanoma patients, metastasis occurs relatively early in the disease and quite frequently [1]. Patients diagnosed with brain metastasis have a short overall survival [3] and systemic therapeutic options for such patients are poor and only beneficial in a limited group of patients [4].

The brain microenvironment is unique with respect to anatomy, resident cells (e.g., astrocytes, microglia), molecular milieu and the immune landscape [5,6].

Tumor cells that disseminated to the brain microenvironment engage in a cross talk with its components thereby acquiring a phenotype which is adapted to this microenvironment. The brain-invading cells in turn, contribute to the remodeling of the brain microenvironment and its establishment as a hospitable accommodation. 

The interactions of the brain-metastasizing cells with brain microenvironmental cells (BMC) may lead either to further metastatic progression or to the elimination of metastasis [1,7,8,9].

Elucidating the pathways leading to metastatic progression or to its inhibition is an essential pre-requisite for the identification of new therapeutic targets and of novel anti-metastatic treatment modalities. 

Metastasis (including brain metastasis) can be driven or inhibited by three major types of signaling factors—tumor-intrinsic factors, microenvironmental factors and downstream factors. The downstream factors are generated by interactions between the metastasizing tumor cells and the metastatic microenvironment.

To approach the identification of drivers and inhibitors of melanoma brain metastasis (MBM) we developed a xenograft model of human MBM in nude mice. Local (cutaneous) tumor and brain metastatic variants were developed from single human melanomas. Each pair of variants originated thus from an identical genetic lineage [10]. Comparing transcriptomic [10], proteomic [11] and epigenomic [12] profiles of these variants, we identified and characterized a number of tumor-intrinsic and microenvironmental signaling factors as well as downstream factors. These three types of factors were involved in driving or inhibiting MBM formation, survival and preservation [7,8,9,10,13,14,15,16,17,18].

In the present study we explored, further, the functional significance of interactions between brain-metastasizing melanoma cells and microenvironmental cells of the brain. We found that the levels of granulocyte-monocyte colony stimulating factor (GM-CSF) in the metastatic microenvironment (MME) of the brain were upregulated as a result of these interactions. This cytokine promoted or restrained the progression of melanoma cells towards metastasis.

## 2. Materials and Methods

### 2.1. Cell Culture

The development, culturing and maintenance of human cutaneous melanoma variants YDFR.C and DP.C and human MBM variants YDFR.CB3 and DP.CB2 were previously described [10,13]. The original cell lines from which these variants were established were kindly provided by Michael Micksche (YDFR, Department of Applied and Experimental Oncology, Vienna University, Austria) and Dr. Dave S.B. Hoon (DP-0574-Me, Department of Translational Molecular Medicine, John Wayne Cancer Institute, Saint John’s Health Center Providence Health Systems, Santa Monica, CA, USA). mCherry-expressing melanoma cells (YDFR.CB3 and DP.CB2) harboring the pQCXIP-mCherry plasmid were constructed, cultured and maintained as previously described [9]. Immortalized human brain microvascular endothelial cells (hCMEC/D3, BEC) were kindly provided by Dr. Clara Nahmias and Prof. Pierre-Olivier Couraud (Inserm, U1016, Institute Cochin, Paris, France) and were maintained as previously described [19]. Human astrocytes (HA; Cat# 1800, lot# 9063, ScienCell Research Laboratories, Carlsbad, CA, USA) were maintained as previously described [16]. Immortalized human microglia-SV40 cell line (RRID: CVCL_YN91, Cat# T0251, lot# RZ825016, ABM, Milton, ON, Canada) was maintained as previously described [14]. The identity of all cell lines used was authenticated using STR. 0.5% FCS supplemented medium was used for starvation in all the experiments. Cells were routinely cultured in humidified air with 5% CO2 at 37 °C.

### 2.2. Preparation of Melanoma- or Brain Cell-Conditioned Medium

Melanoma cells or brain cells (microglia, brain endothelial cells (BEC) and astrocytes) were cultured for 24 h, then starved for additional 24 h. Melanoma-conditioned medium (MCM), brain endothelial cell-conditioned medium (BEC-CM), microglia-conditioned medium (MG-CM) or astrocyte-conditioned medium (HA-CM) was collected, centrifuged for 5 min at 1400 rpm and filtered (0.45 μm, Whatman GmbH, Dassel, Germany).

### 2.3. ELISA Assay

For the estimation of basal GM-CSF levels, cells were plated and grown in 0.5% FCS supplemented starvation medium for 24 h. For CM-treatments, cells were plated and stimulated with CM or with starvation media as control for 4 h, then washed and starved for 24 h. For cytokine treatments, cells were stimulated with recombinant cytokines for 24 h. Alternatively, cells were co-stimulated with CM and cytokines for 4 h, then starved for 24 h. Cytokines used were—recombinant human interleukin-1α (hIL-1α, 1–50 ng/mL) and recombinant human tumor necrosis factor-α (rhTNF-α, 1−50 ng/mL) (PeproTech, Rocky Hill, NJ, USA).

The supernatants were then collected, centrifuged, filtered and 15-fold (melanoma supernatants) or 30-fold (brain cell supernatants) concentrated at 4000× *g* using Amicon® Ultra-15 centrifugal filter units (Merck Millipore, Burlington, MA, USA) for 1 h. The fraction (MW > 3 kDa) was used to determine the extracellular levels of GM-CSF by ELISA according to manufacture instructions using the human GM-CSF DuoSet (R&D systems, Minneapolis, MN, USA).

### 2.4. Downregulation of GM-CSF Expression

The downregulation of GM-CSF was constructed using pGIPZ vectors (Thermo Fisher Scientific, ABgene, Germany) containing shRNA sequences targeting human *CSF2* mRNA (NM_000758.4). For the preparation of melanoma GM-CSF knocked-down cells, a combination of two vectors was used (V3LHS_374948 and V3LHS_374949) to transfect YDFR.CB3 and DP.CB2 cells (shCSF2). The cells were produced as previously described [13]. A sh-non-silencing pGIPZ vector (RHS4531) was used as a negative control (shControl). All plasmids used were containing a GFP-tag. Transfected cells were selected using 1 µg/mL puromycin (InVivoGen, San Diego, CA, USA).

### 2.5. Adhesion to Brain Endothelial Cells 

Adhesion of melanoma cells to BEC was performed as previously described [16] with minor modification. Briefly, the cells were stimulated with 10 ng/mL recombinant human GM-CSF (rhGM-CSF) (PeproTech, Rocky Hill, NJ, USA) in starvation medium or cultured in starvation medium as control for 24 h prior to the incubation of melanoma cells upon the BEC monolayer. Adhesion of mCherry-expressing cells was measured at wavelength of 590/645. To obtain the percentage of adherent cells, the optical density (OD) of the adherent cells was divided by the OD of the total cells plated. 

### 2.6. Transendothelial Migration Through a Blood-Brain Barrier Model

Transendothelial migration assays were performed as previously described [13] with modifications. For mCherry-melanoma transendothelial migration assays, 1 × 10^5^ cells were loaded onto BEC monolayer-seeded transwells (8 µm; Corning Costar Corp.) with or without 10 ng/mL rhGM-CSF. Alternatively, melanoma or BEC cells were stimulated with rhGM-CSF (separately) for 24 h prior to the loading of melanoma onto BEC monolayer-seeded transwells. Cells were allowed to migrate for 24 h. For *CSF2*-silenced melanoma migration assays, 1 × 10^5^ cells were loaded onto BEC monolayer-seeded transwells and allowed to migrate for 24 h. Fixation, imaging of transwells and respective biostatistical analysis was performed as previously described [13].

### 2.7. Immunodetection of Proteins by Western Blot

BEC were plated and stimulated with 30 ng/mL rhGM-CSF for 2, 6, 24 or 48 h. As control, cells were grown without rhGM-CSF for an equal amount of time. The cells were washed twice with ice-cold physiological phosphate-buffered saline (PBS) and lysed as previously described [17]. Proteins were separated on 4–12% Bis–Tris gels (Thermo Fisher Scientific, ABgene, Germany) and transferred onto nitrocellulose membranes. The membranes were blocked at room temperature with 3% BSA diluted in TBS–Tween for 1 h. The following primary Abs were used—anti-claudin-5 (A-12) Ab (Cat# sc-374221, RRID:AB_10988234, Santa Cruz Biotechnology, Dallas, TX, USA), anti-occludin (E5) Ab (Cat# sc-133256, RRID:AB_2156317, Santa Cruz Biotechnology), anti-β-tubulin Ab - Loading Control (Cat# ab-6046, RRID:AB_2210370, Abcam, Cambridge, UK) and anti-zonula occludens-1 (ZO-1) (H-300) Ab (sc-10804, RRID:AB_2205514, Santa Cruz Biotechnology, Dallas, TX, USA). As secondary Abs, horseradish peroxidase-conjugated donkey anti-mouse Ab or goat anti-rabbit Ab (1:10,000, Jackson ImmunoResearch Laboratories, West Grove, PA, USA) were used. The gel bands were visualized by chemiluminescence ECL reactions (Merck Millipore). The processing of bands intensity was performed with ImageQuant TL Version 8.1 (GE Healthcare Life Sciences, Chicago, IL, USA). Each experiment was repeated 3–5 times.

### 2.8. Animals

Male athymic nude mice (BALB/c background) were purchased from Harlan Laboratories Limited (Jerusalem, Israel). The mice were housed and maintained in laminar flow cabinets under specific pathogen-free conditions in the animal quarters of Tel-Aviv University and in accordance with current regulations and standards of the Tel-Aviv University Institutional Animal Care and Use Committee. The project identification code: 04-16-044; date of approval: 11/07/2016. The mice were used when they were 7–8 weeks old.

### 2.9. Orthotopic Inoculation of Tumor Cells

To generate subcutaneous (SC) tumors, mice (*n* = 8 in each group) were inoculated SC with 1 × 10^6^ melanoma cells in 100 μL of 5% FCS RPMI-1640 medium as previously described [10]. To test the tumorigenic properties of derived cell lines, SC tumors were measured once a week using a caliper. Tumor volume was obtained by the ellipsoid volume calculation formula Tumor volume =0.5×(length × width × width) as previously described [20].

### 2.10. Intracardiac Inoculation of Tumor Cells

For intracardiac (IC) inoculation, cells were harvested by trypsinization and transferred into RPMI-1640 medium supplemented with 5% FCS. 

Prior to IC inoculation, nude mice (*n* = 8 in each group) were anesthetized by ketamine (100 mg/kg body mass) and xylazine (10 mg/kg body mass) (Kepro, Deventer, The Netherlands) administered intraperitoneally. Using a small animal ultrasound device (Vevo 770 High-Resolution In Vivo Micro-Imaging System; VisualSonics, Toronto, Canada), 5 × 10^5^ cells in 50 μL of 5% FCS RPMI-1640 medium were inoculated into the left ventricle of the heart, using a 29-gauge needle. Mice were sacrificed, brains were dissected out and immediately cryopreserved at −70 °C until used for RNA extraction. 

### 2.11. RNA Preparation and Reverse Transcription Droplet Digital PCR (RT-ddPCR)

Total cellular RNA was extracted from mice brains using EZ-RNA Total RNA Isolation Kit (Biological Industries, Kibbutz Beit Haemek, Israel). RNA concentrations were determined by the absorbance at 260 nm and quality control standards were A260/A280 = 1.8–2.0. 1 μL of each RNA sample was used for cDNA synthesis using the qScript cDNA Synthesis Kit (Quantabio, Beverly, MA, USA) according to the manufacturer’s instructions. The detection of human cDNA was conducted with QX200 ddPCR System (BioRad, Philadelphia, PA, USA). The reaction mix was prepared with ddPCR Supermix for Probes (BioRad, Philadelphia, PA, USA), 1 μL of cDNA and probe assay consisting of unlabeled PCR primers and a labeled fluorescent probe. The following primers were used—β2 microglobulin (β2m), Human, tagged with FAM (unique Assay ID: dHsaCPE5053100, BioRad, Philadelphia, PA, USA) and β2m, Mouse, tagged with HEX (unique Assay ID: dMmuCPE5124781, BioRad, Philadelphia, PA, USA). Each run included a positive control (cDNA from human melanoma cell culture), negative control (cDNA from naïve mouse brain) and no template control. Droplet generation, transfer of droplets, plate sealing and PCR reaction conditions were as described by the manufacturer. The processing of PCR products was performed with QuantaSoft Version 1.7.4 (BioRad, Philadelphia, PA, USA). 

### 2.12. mRNA Sequencing Analysis 

Cells were plated and grown in starvation media for 24 h with or without 10 ng/mL rhGM-CSF. Plates were washed with cold PBS and RNA was extracted using miRNeasy mini kit (Qiagen, Valencia, CA, USA). Concentration of purified total RNA was measured using the Quant-iT RiboGreen RNA assay (Life Technologies, Carlsbad, CA, USA) and RNA quality was assessed by the RNA ScreenTape assay on the Agilent TapeStation 2200 (Agilent Technologies, Santa Clara, CA, USA). Using 1ug of high quality (RIN > 7.0) total RNA, mRNA libraries were prepared from 3 independent repeats for each treatment with the NEXTflex Rapid Directional mRNA-Seq Kit (Bioo Scientific, Austin, TX, USA). Quality and quantity of final libraries were assessed by High Sensitivity D1000 assay (Agilent Technologies) and Qubit dsDNA HS assay (Life Technologies, Carlsbad, CA, USA), respectively. Libraries were pooled and sequenced on an Illumina NextSeq 550 (Illumina Inc, San Diego, CA, USA) using 76bp paired-end reads.

Raw RNA sequencing reads were checked for overall quality and filtered for adapter contamination using Trimmomatic (version 0.36) [21]. The filtered reads were then mapped to the GENCODE comprehensive gene annotation reference set (version 19) using the STAR aligner (version 2.4.2a) [22] with default parameters. Read counts for each feature were generated using the “--quantModeGeneCounts” function in STAR. Significantly differentially expressed genes were identified using ANOVA with a significance threshold of fold change (FC) < −1.5 or FC > 1.5 and *p*-value ≤ 0.05.

### 2.13. Biostatistical Analysis

Data were analyzed using Student’s t test and considered significant at *p*-values ≤ 0.05. Bar graphs represent mean and standard deviation (SD) or standard error of the mean (SEM) across multiple independent experimental repeats.

## 3. Results

### 3.1. Melanoma-Derived Soluble Factors (MCM) Enhance GM-CSF Secretion from Brain Microenvironmental Cells

**Figure 1:**
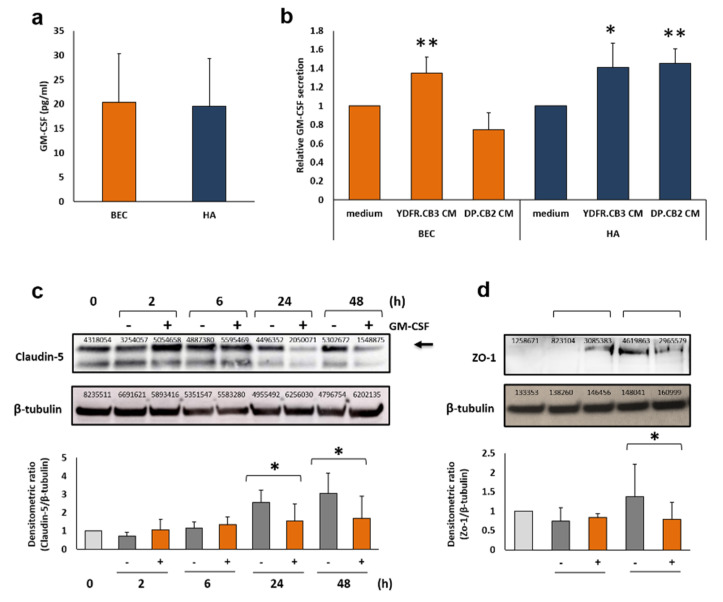
Melanoma-secreted factors stimulate granulocyte-monocyte colony stimulating factor (GM-CSF) secretion from brain endothelial cells (BEC) and astrocytes. Alteration of tight junction protein levels in BEC. (**a**,**b**) Extracellular levels of GM-CSF were determined by ELISA analysis. (**a**) 1 × 10^6^ BEC or astrocytes (HA) were cultured for 24 h. The bars represent GM-CSF levels in 30-fold concentrated supernatants (pg/mL). (**b**) 1 × 10^6^ BEC or astrocytes were treated with YDFR.CB3 or DP.CB2 CM for 4 h, then cultured in starvation medium for 20 h. Treatment with 0.5% FCS containing medium was used as control. The bars represent the relative GM-CSF levels normalized to control. All graphs represent an average of at least three independent experiments ±SD. * *p* < 0.05, ** *p* < 0.01. (**c**) BEC were stimulated with 30 ng/mL rhGM-CSF for 2, 6, 24 and 48 h (+). Control, untreated cells were grown in starvation medium for 0, 2, 6, 24 and 48 h (−). Western blot was applied to detect claudin-5 (23 kDa, marked with an arrow) and β-tubulin (55 kDa) for loading control in the cell culture lysates. Representative images are shown. (**d**) BEC were stimulated with 30 ng/mL rhGM-CSF for 2 and 6 h (+). Control, untreated cells were grown in starvation medium for 0, 2 and 6 h (−). Western blot was applied to detect ZO-1 (220 kDa) and β-tubulin in the cell culture lysates. Representative images are shown. The intensity of claudin-5 and ZO-1 signal was divided by the intensity of β-tubulin signal. The relative intensity was then normalized to t = 0. Graphs represent the average ±SD of at least three independents repeats. * *p* < 0.05.

GM-CSF is secreted from unstimulated BEC and from astrocytes but not from microglia (Figure 1a). Soluble factors derived from YDFR.CB3 cells, the brain metastatic variant of the human YDFR melanoma cell line [10], significantly increased GM-CSF secretion from BEC and astrocytes by 35% and 40%, respectively (Figure 1b). Soluble factors derived from DP.CB2 cells, the brain metastatic variant of the human DP-0574-Me melanoma cell line [13], significantly increased GM-CSF secretion from astrocytes by 45% but did not affect GM-CSF secretion from BEC. Microglia treated with YDFR.CB3 or with DP.CB2 MCM did not secrete GM-CSF.

### 3.2. The Effects of GM-CSF on Brain Endothelial Cells

Tumor-endothelium interactions are pivotal in brain metastasis formation. We therefore evaluated the effects of GM-CSF on the gene expression profile of BEC and on the integrity of several of their tight junction (TJ) components being highly involved in transendothelial migration.

#### 3.2.1. GM-CSF Alters the Gene Expression Profile of BEC

We compared the gene expression profile of rhGM-CSF-activated BEC to that of untreated BEC. The results (Table 1) indicated that in general GM-CSF-activated genes promote metastasis progression by positively regulating transendothelial migration and angiogenesis.

- Transendothelial migration related genes
  1. The upregulated gene adenylate cyclase 10 (ADCY10, FC = 3.01) regulates endothelial stiffness and protects endothelial barrier function under inflammatory and hypoxic conditions [23]. Adenylate cyclase inhibition blocked transendothelial migration [24].
  2. The downregulated gene interleukin-37 (IL-37, FC = −2.36) inhibits inflammatory response by suppressing the TLR2-NF-κB-ICAM-1 pathway in coronary artery endothelial cells and is possibly involved in the adhesion and transmigration of neutrophils through such endothelial cells [25]. Additionally, IL-37 promotes endothelial activation and angiogenesis [26].
- Angiogenesis related genes
  1. The upregulated gene carbonic anhydrase 9 (CA9, FC = 4.18) induces endothelial migration and angiogenesis in tumors [27].
  2. The upregulated gene serum/glucocorticoid regulated kinase 2 (SGK1, FC = 2.84) is required for endothelial cell migration and angiogenesis [28].
  3. The downregulated gene ephrin B3 (EFNB3, FC = −3.99) supports endothelial cell survival and its silencing decreases tumor vascularization and growth in a glioblastoma xenograft model [29].
  4. The downregulated gene RUNX family transcription factor 3 (RUNX3, FC = −2.33) contributes to endothelial-to-mesenchymal transition and endothelial cell dysfunction. RUNX3 downregulation reduced endothelial cell migration and promoted angiogenesis [30].

#### 3.2.2. GM-CSF Down-Regulates the Expression of the Endothelial Tight Junction Proteins Claudin-5 and Zonula Occludens-1

TJs regulate permeability of the blood-brain barrier (BBB) [31]. In light of findings that GM-CSF modulates TJ components claudin-5 [32] and zonula occludens-1 (ZO-1) [33] thereby altering transendothelial migration of mouse monocytes, we asked if human GM-CSF would also induce similar effects in human brain endothelial cells. rhGM-CSF was added to cultured BEC for 2, 6, 24 and 48 h. A significant decrease in claudin-5 levels in GM-CSF treated BEC was observed 24 and 48 h post stimulation (Figure 1c). Similarly, expression levels of ZO-1 decreased in GM-CSF treated BEC 6 h post stimulation (Figure 1d). GM-CSF did not alter the levels of another TJ related protein, occludin (data not shown). These results suggest that GM-CSF has the capacity to control the permeability of melanoma cells through the BBB by downregulating the expression of TJ components claudin-5 and ZO-1.

### 3.3. GM-CSF is Secreted from Human Melanoma Cells

**Figure 2:**
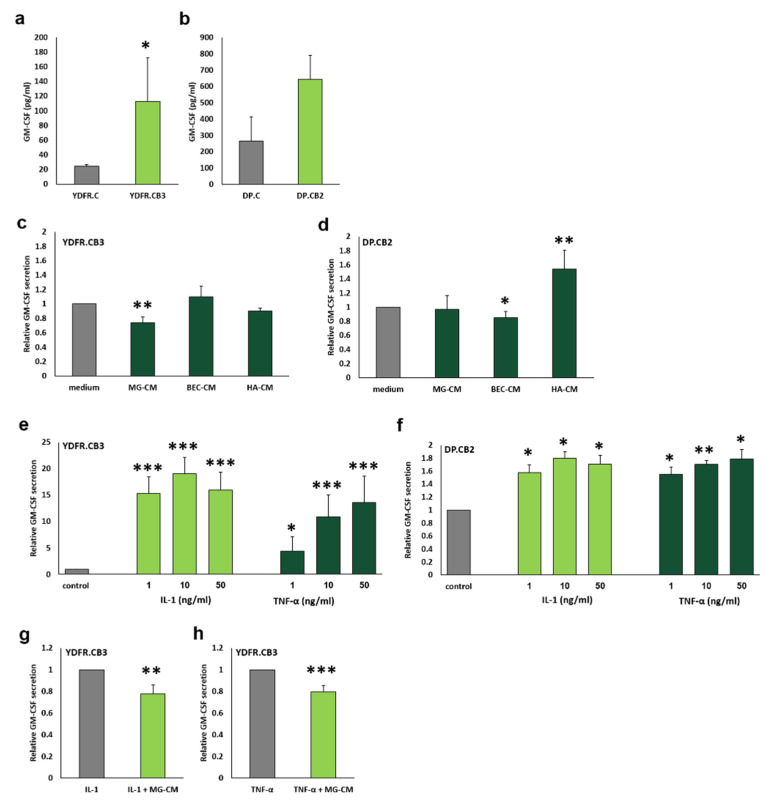
Regulation of melanoma-derived GM-CSF by brain microenvironment-secreted factors. (**a**–**h**) Extracellular levels of GM-CSF were estimated by ELISA analysis. (**a**,**b**) 5 × 10^5^ YDFR.C, YDFR.CB3 (**a**), DP.C and DP.CB2 (**b**) cells were cultured for 24 h. The bars represent GM-CSF levels in 15-fold concentrated supernatants (pg/mL). (**c**,**d**) 5 × 10^5^ YDFR.CB3 (**c**) or DP.CB2 (**d**) cells were treated with microglia-CM (MG-CM), brain endothelial cell-conditioned medium (BEC-CM) or astrocyte-conditioned medium (HA-CM). After 4 h, cells were cultured in starvation medium for 20 h. Treatment with starvation medium was used as control (medium). (**e**,**f**) 5 × 10^5^ YDFR.CB3 (**e**) and DP.CB2 (**f**) cells were simulated with rhIL-1α (1, 10 and 50 ng/mL) or rhTNF-α (1, 10 and 50 ng/mL) for 24 h. Unstimulated cells were used as control. Note: The scale of the two graphs is different. (**g**,**h**) 5 × 10^5^ YDFR.CB3 cells were stimulated with either rhIL-1α (**g**) or rhTNF-α (**h**) (10 ng/mL) diluted in MG-CM (rhIL1 + MG-CM, rhTNF-α + MG-CM) or in starvation medium as control (rhIL1-1 α, rhTNF-α) for 4 h, then cultured in starvation medium for 20 h. For each of the experiments, the bars represent the relative GM-CSF levels normalized to control. All graphs represent an average of at least three independent experiments ±SD. * *p* < 0.05, ** *p* < 0.01, *** *p* < 0.001.

Cell lines derived from 2 different melanomas YDFR and DP-0574-Me (DP) were employed in this study. Both local (cutaneous) and brain metastatic variants from these cell lines (YDFR.C, DP.C and YDFR.CB3, DP.CB2 respectively) secreted GM-CSF but in different amounts (Figure 2a,b). The overall GM-CSF secretion from the DP variants was higher than that secreted from the YDFR variants. The metastatic YDFR.CB3 cells secreted higher amounts of GM-CSF than the cutaneous YDFR.C cells. The same trend was found for the DP cell line, as the metastatic DP.CB2 cells secreted higher amounts of GM-CSF than the cutaneous DP.C cells, though statistically insignificant.

### 3.4. Brain-Derived Soluble Factors Alter GM-CSF Secretion from Brain-Metastasizing Melanoma Cells

We next determined if brain microenvironmental cells are capable of modulating GM-CSF secretion from melanoma cells. Whereas BEC- and astrocyte-derived soluble factors did not alter GM-CSF secretion from the brain metastatic variant YDFR.CB3, microglia-derived soluble factors decreased GM-CSF secretion from these cells by ~25% (Figure 2c). Soluble factors from brain-microenvironmental cells elicited a different pattern of GM-CSF secretion from DP.CB2 cells. While BEC-derived soluble factors decreased GM-CSF secretion by 25%, astrocytes-derived factors increased GM-CSF secretion by 54%. Microglia-derived soluble factors did not alter GM-CSF secretion (Figure 2d).

### 3.5. IL-1α and TNF-α Differentially Influence GM-CSF Secretion from Melanoma Cells

The expression of the inflammatory cytokines IL-1α and TNF-α increases significantly in CNS pathologies [34,35]. Increased levels of these cytokines in the brain are associated with increased BBB permeability [34]. In order to determine if these 2 cytokines play a regulatory role in GM-CSF secretion from melanoma cells we treated YDFR.CB3 and DP.CB2 cells with rhIL-1α and rhTNF-α. These cytokines strongly enhanced GM-CSF secretion from YDFR.CB3 and DP.CB2 cells (Figure 2e,f). The enhancement of GM-CSF secretion from YDFR.CB3 cells by rhIL-1α or rhTNF-α treatment was inhibited by ~20 % when melanoma cells were treated with rhIL-1α or rhTNF-α mixed with microglia conditioned medium (Figure 2g,h). GM-CSF secretion from DP.CB2 cells following a co-incubation with the two cytokines in combination with MG-CM was not measured since MG-CM did not affect GM-CSF secretion from DP.CB2 cells. These results indicate that in addition to factors such as IL-1α or TNF-α that stimulate the secretion of GM-CSF from melanoma cells, the microglial secretome contains factors that inhibit GM-CSF secretion from melanoma cells.

### 3.6. Varied Responses of YDFR and DP Cells to GM-CSF-Mediated Activation

Based on the results reported above we hypothesized that GM-CSF reprograms the metastatic phenotype of melanoma cells and that the response of melanoma cells of different individuals to signals mediated by this cytokine are not uniform.

#### 3.6.1. GM-CSF Differentially Affects the Interaction of Melanoma Cells with BEC 

**Figure 3:**
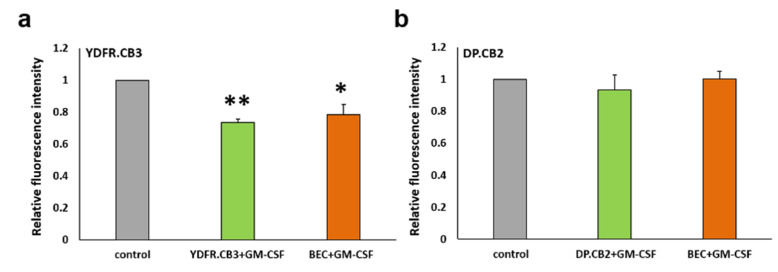
GM-CSF interferes with the interaction of certain metastatic melanoma cells with brain endothelial cells (BEC). (**a**,**b**) mCherry-expressing melanoma cells YDFR.CB3 (**a**) or DP.CB2 (**b**) were stimulated with 10 ng/mL rhGM-CSF for 24 h before seeded on top of BEC monolayer and incubated for 30 min, to allow adhesion to occur. Alternatively, unstimulated melanoma cells were seeded on rhGM-CSF-stimulated BEC. Adhesion assay of unstimulated melanoma and BEC was performed as control. The fluorescence signal of labeled cells was measured before and after removal of non-adherent cells. The bars represent the relative florescence intensity normalized to control. Each experiment consisted of 6 repeats for each treatment. The graph represents an average of three independent experiments ±SD. * *p* < 0.05, ** *p* < 0.01.

Adhesion of circulating cancer cells to endothelial cells is an initial step in metastatic colonization at a metastatic organ site [36]. Since MCM increases the secretion of GM-CSF from BEC we asked if such an increase would influence the adhesion of melanoma cells to BEC. Prior to the adhesion assay, mCherry-expressing YDFR.CB3 and DP.CB2 cells were cultured for 24 h in the presence of rhGM-CSF. BEC were cultured similarly. Untreated cells served as controls. Co-adhesion of the two cell types was then evaluated. The adherence of GM-CSF pretreated YDFR.CB3 cells to control BEC was decreased by 25% (*p* < 0.01). Similarly, the adhesion of control YDFR.CB3 cells co-cultured with GM-CSF-treated BEC was decreased by 20% (*p* < 0.05) (Figure 3a). No significant difference was found between the adhesive capacity of GM-CSF pre-treated or untreated DP.CB2 cells to GM-CSF-treated or control BEC (Figure 3b). 

**Figure 4:**
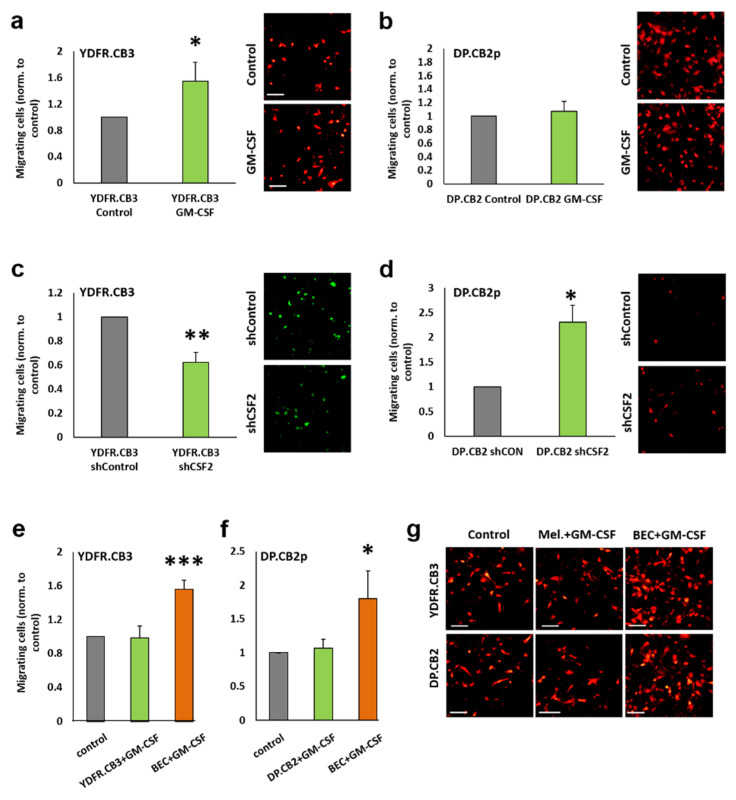
GM-CSF differentially mediates melanoma transendothelial migration. (**a**,**b**) mCherry-expressing YDFR.CB3 (**a**) and DP.CB2 (**b**) cells migrated through brain endothelial cells(BEC) in the absence (Control) or presence (GM-CSF) of 10 ng/mL rhGM-CSF. (**c**,**d**) Transendothelial migration of CSF2-silenced YDFR.CB3 (**c**) and DP.CB2 (**d**) cells (shCSF2) and their corresponding control cells (shControl). (**e**–**g**) Untreated mCherry-expressing YDFR.CB3 (**e**,**g**) and DP.CB2 (**f**,**g**) cells migrated through untreated BEC (Control); 10 ng/mL rhGM-CSF pre-treated melanoma cells migrated through untreated BEC (melanoma + GM-CSF); untreated melanoma cells migrated through 10 ng/mL rhGM-CSF pre-treated BEC (BEC + GM-CSF). Representative images for each treatment are shown. Bar: 100 µm. For each of the experiments, the bars represent the average number of migrating cells/field normalized to control. Each experiment consisted of 2–3 repeats for each treatment, in each 6–8 fields were imaged and counted. The graph represents an average of 3–4 independent experiments ±SD. * *p* < 0.05, ** *p* < 0.01, *** *p* < 0.001.

The impact of GM-CSF on transendothelial migration was investigated next. An experimental BBB model consisting of cell-permeable transwells seeded with BEC was employed. The transmigration of mCherry-expressing YDFR.CB3 and DP.CB2 cells through the BEC layer was evaluated in the presence or absence of rhGM-CSF. Whereas GM-CSF enhanced the transmigration of the YDFR.CB3 cells it did not enhance the transendothelial migration of DP.CB2 cells (Figure 4a,b). 

To further validate these results, we generated GFP-expressing YDFR.CB3 and DP.CB2 cells in which GM-CSF was silenced by specific shRNA (shCSF2). Control cells were infected with a non-silencing shRNA (shControl). The plasmids used contained a GFP tag. GM-CSF silencing did not affect cell viability (data not shown). These experiments supported the findings that GM-CSF positively regulates the transendothelial migration of YDFR.CB3 cells; the transmigration of YDFR.CB3-shCSF2 cells was reduced compared to control cells (Figure 4c). Similar experiments performed with DP.CB2-shCSF2 cells yielded opposite results, as the transmigration of DP.CB2-shCSF2 cells was higher than that of the control cells (Figure 4d). In order to identify which of the two interacting cells (melanoma or BEC) responded to GM-CSF under these conditions, mCherry-expressing YDFR.CB3 cells, DP.CB2 cells or BEC were treated with rhGM-CSF prior to measuring transendothelial migration. Whereas GM-CSF pre-treatment of BEC led to increased transmigration of YDFR.CB3 as well as DP.CB2 melanoma cells, the migration of pre-treated melanoma cells through untreated BEC was similar to that of untreated control melanoma cells (Figure 4e,f) These results indicate that GM-CSF may enhance the transendothelial migration of cells originating in some melanomas by reprograming BEC. GM-CSF-mediated enhanced transendothelial migration of melanoma cells occurs only through reprogrammed (but not naïve) BEC.

#### 3.6.2. GM-CSF Either Promotes or Inhibits Local Tumor Formation by Melanoma Cells

**Figure 5:**
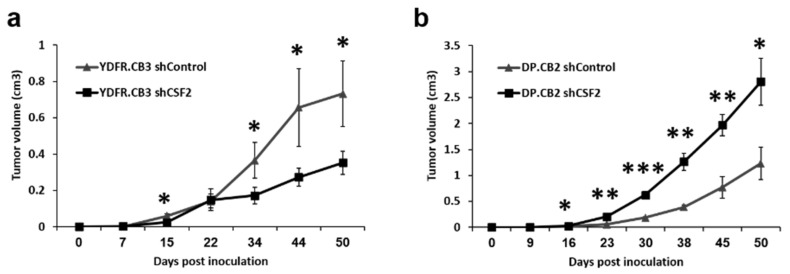
GM-CSF promotes or inhibits local tumor formation by different melanoma cells. (**a**,**b**) Nude mice were inoculated SC with GM-CSF silenced (shCSF2) or control (shControl) YDFR.CB3 and DP.CB2 cells (*n* = 8 in each group). Tumor dimensions of cutaneous tumors established from YDFR.CB3 (**a**) and DP.CB2 (**b**) cells were measured using a caliper and volume was obtained as described in Materials and Methods. The average tumor volume + SEM is presented. * *p* < 0.05, ** *p* < 0.001, *** *p* < 0.0001.

Adhesion of circulating cancer cells to endothelial cells is an initial step in metastatic colonization at a metastatic organ site [36]. Since MCM increases the secretion of GM-CSF from BEC we asked if such an increase would influence the adhesion of melanoma cells to BEC. Based on results demonstrating that GM-CSF differentially shapes the malignant phenotype of melanoma cells in vitro, we next asked if GM-CSF plays a role in the formation of local xenografted melanoma tumors. Nude mice (*n* = 8 per group) were inoculated orthotopically (subcutaneously) with shCSF2 or shControl YDFR.CB3 and DP.CB2 cells. Volume measurements of local tumors demonstrated that the tumors formed by YDFR.CB3 shControl cells were significantly larger than tumors originating from YDFR.CB3 shCSF2 cells (Figure 5a). This indicated that GM-CSF promoted the growth of these melanoma cells. Conversely, tumors originating from the DP.CB2 shControl cells were significantly smaller than tumors originating from control DP.CB2 shCSF2 cells (Figure 5b). This demonstrated that GM-CSF inhibited local tumor formation by DP.CB2 cells. 

#### 3.6.3. GM-CSF May Impact Melanoma Brain Metastasis

We asked if expression levels of GM-CSF by melanoma cells affect the formation of brain metastasis. To answer this question, we inoculated, via the intracardiac route, nude mice with control YDFR.CB3 and DP.CB2 cells or with cells in whom GM-CSF was knocked-down. The mice were sacrificed six weeks later. Quantification of human and mouse RNA in the brains was performed using RT-ddPCR with human and mouse β2m primers, as a measure of the presence of human melanoma cells in mouse brain [10]. The median value of human/mouse β2m mRNA expression in the brains in the group of mice inoculated with GM-CSF knocked-down YDFR.CB3 cells was lower than the median value of human/mouse β2m mRNA expression in the brains of mice inoculated with YDFR.CB3 shControl cells (0.758 vs 1.14). GM-CSF knock-down in DP.CB2 cells yielded opposite results. The median value of human/mouse β2m mRNA expression in the brains in the group of mice inoculated with GM-CSF silenced DP.CB2 cells was higher than the median value of human/mouse β2m mRNA expression in the brains of mice inoculated with DP.CB2 shControl cells (4.14 vs 1.37). These results are compatible with the tumorigenicity-modifying function of GM-CSF reported above. Taken together the results of this study show (Table 2) that:

1. GM-CSF exerts regulatory functions on the metastatic microenvironment of the brain.
2. GM-CSF impacts differently the malignant phenotype of melanoma cells from different patients; augmenting the malignancy of one melanoma while restraining the malignancy of another.

## 4. Discussion

GM-CSF, a hematopoietic cytokine, produced by T cells, macrophages and a variety of non-lymphoid/myeloid stroma cells, is a key factor in the maturation, differentiation, proliferation and activation of myeloid cells such as macrophages, granulocytes, dendritic cells and so forth. GM-CSF has inflammatory and immune regulatory functions and as such is an essential constituent of various immune responses [37].

GM-CSF being a crucial factor in anti-tumor immunity [38,39], is an important constituent of various anti-cancer immunotherapy trials. Preclinical as well as clinical studies yielded beneficial responses in certain populations of cancer patients or in experimental animals and harmful responses in others [40,41].

Summarizing the state of the art regarding clinical trials in which GM-CSF has been used as an adjuvant in many different clinical trial settings in melanoma patients, Hoeller noted that evidence for clinical efficacy of GM-CSF is controversial and that the optimal treatment regimen and effectiveness of such treatment in patients with advanced melanoma has to be worked out [42]. 

Studies performed in our lab aim to identify drivers or inhibitors of melanoma brain metastasis. To reach this goal we analyze the interactions of brain-metastasizing melanoma cells with brain microenvironmental cells [7,8,9]. The major conclusion derived from these studies was that the cross-talk between brain-metastasizing melanoma cells and microenvironmental cells residing in the brain determine metastasis formation in this organ [43]. 

In the present study we found that melanoma-brain interactions impact the expression of GM-CSF and its secretion from both the melanoma cells as well as brain cells.

GM-CSF altered the gene-expression profile of brain endothelial cells rendering them supportive for metastatic progression. Indeed, GM-CSF downregulated the expression of TJ components thereby increasing BBB permeability.

Microglia that did not secrete GM-CSF, did inhibit, to a certain extent its secretion from melanoma cells showing that microglia cells are involved, indirectly, in establishing the microenvironmental levels of this cytokine. Microglia-derived IL-1α and TNF-α were found to upregulate GM-CSF secretion from melanoma cells. This demonstrates that microglia, like many other cells, secrete, simultaneously, factors that may exert opposite effects on melanoma progression. The bioactivity of such a mixture of agonists and antagonists is determined by a balance between the two. In this case microglia-derived factors that inhibit GM-CSF secretion from melanoma cells were dominant.

A major result of this study is that melanoma cells from 2 different cell lines differed from each other in their response to GM-CSF (Table 2). Whereas GM-CSF promoted the in vitro malignancy phenotype and the in vivo local tumorigenicity and brain metastasis of YDFR.CB3 cells, it either did not influence the malignancy of DP.CB2 cells or even reduced it. 

These results may constitute a relevant example of inter-tumor heterogeneity [44] reported recently by us to occur in melanoma cells derived from 4 individual melanoma patients [11]. Cutaneous and brain metastatic variant pairs from these melanomas, sharing the same genetic ancestry, were subjected to proteome profiling aiming to identify shared molecular pathways leading to brain metastasis. This analysis, although revealing a large variety of proteins differentially expressed by local and brain metastatic variants, did not identify any protein that characterizes the transition from cutaneous melanoma to brain metastasis which is shared by the 4 melanomas. This inter-melanoma heterogeneity may be the basis for the differential response of melanoma patients to GM-CSF-associated therapy [42,43,44,45,46].

Intra-melanoma heterogeneity [47] may have also played a role in the opposing effects of GM-CSF on the malignancy phenotype of the 2 melanoma cells investigated in the present study. The YDFR.CB3 population may be composed of a majority of cells responding by a heightened malignancy to GM-CSF mediated signaling whereas the DP.CB2 population may be composed of a majority of cells which are refractory to such signals. Genomic studies should provide the mechanisms underlying the contradictory response of melanomas (increased or decreased malignancy) to GM-CSF-mediating signaling.

We hypothesize that patient-derived melanomas may express either the YDFR.CB3 or DP.CB2 phenotype with respect to genomic, transcriptomic and functional responses to GM-CSF. 

In vitro responses of melanoma cells to GM-CSF may be developed to a predictive biomarker to better assess the response of individual melanoma patients to GM-CSF treatment thereby providing a tool to segregate melanoma patients who will benefit from GM-CSF therapy from those who will not.

## Figures and Tables

**Table 1:** Differentially expressed genes in rhGM-CSF-stimulated BEC vs. untreated control cells.

Gene Symbol	Gene Name	FC	*p*-Value
Upregulated			
LYPD3	LY6/PLAUR domain containing 3	7.62	0.000944
HPD	4-hydroxyphenylpyruvate dioxygenase	5.26	0.000679
CA9	carbonic anhydrase 9	4.18	7.42 × 10^−5^
MIR3124	microRNA 3124	4.02	5.36 × 10^−6^
PNPLA1	patatin like phospholipase domain containing 1	3.84	7.94 × 10^−6^
CGB7	chorionic gonadotropin subunit beta 7	3.71	0.000861
PCDHGA7	protocadherin gamma subfamily A, 7	3.66	0.000385
LINC02310	long intergenic non-protein coding RNA 2310	3.63	0.000158
ADCY10	adenylate cyclase 10	3.01	1.1 × 10^−5^
DIPK2B	divergent protein kinase domain 2B	2.94	2.01 × 10^−5^
SGK2	serum/glucocorticoid regulated kinase 2	2.84	0.000594
GLS2	glutaminase 2	2.82	0.000387
CASKIN1	CASK interacting protein 1	2.62	0.000489
LY6G5C	lymphocyte antigen 6 family member G5C	2.42	0.000438
PRSS27	serine protease 27	2.40	0.000651
ARHGAP40	Rho GTPase activating protein 40	2.38	0.00044
ARHGAP9	Rho GTPase activating protein 9	2.15	0.000554
SLC17A7	solute carrier family 17 member 7	2.12	0.00088
PDF	peptide deformylase, mitochondrial	2.07	0.000363
RBM44	RNA binding motif protein 44	2.02	0.000415
ALLC	allantoicase	2.01	5.31 × 10^−6^
**Downregulated**			
RAB11FIP4	RAB11 family interacting protein 4	−6.13	0.000349
CES4A	carboxylesterase 4A	−4.98	6.78 × 10^−5^
KHDRBS3	KH RNA binding domain containing, signal transduction associated 3	−4.41	0.000557
EFNB3	ephrin B3	−3.99	8.79 × 10^−5^
RPL21P28	ribosomal protein L21 pseudogene 28	−2.80	0.000986
LINC00954	long intergenic non-protein coding RNA 954	−2.71	0.000508
IRS2	insulin receptor substrate 2	−2.53	0.000306
TSHZ3	teashirt zinc finger homeobox 3	−2.51	0.000497
CSGALNACT1	chondroitin sulfate N-acetylgalactosaminyltransferase 1	−2.41	7.75 × 10^−5^
KREMEN1	kringle containing transmembrane protein 1	−2.37	0.000685
IL37	interleukin 37	−2.36	2 × 10^−6^
RUNX3	RUNX family transcription factor 3	−2.33	0.000937
ZFP37	ZFP37 zinc finger protein	−2.06	0.000857

List of 34 down-regulated or up-regulated genes (fold change (FC) < −1.5 or FC > 1.5 and *p*-value ≤ 0.05) in brain endothelial cells exposed to 10 ng/mL rhGM-CSF, compared to untreated cells.

**Table 2:** The impact of GM-CSF on the brain metastatic melanoma cells YDFR.CB3 and DP.CB2.

		YDFR.CB3	DP.CB2
**GM-CSF expression**	Expression in cutaneous vs. MBM ^1^ variants	Higher in the metastatic variant	Higher in the metastatic variant (NS ^2^)
	Expression in MG-CM ^3^-treated MBM	Down-regulation	Not altered
	Expression in BEC-CM ^4^-treated MBM	Not altered	Down-regulation
	Expression in HA-CM ^5^-treated MBM	Not altered	Up-regulation
	IL-1α ^6^ treatment	Up-regulation	Up-regulation
	TNF-α ^7^ treatment	Up-regulation	Up-regulation
	IL-1α + MG-CM treatment	Down-regulation	NA ^8^
	TNF-α + MG-CM treatment	Down-regulation	NA
**in-vitro function of** **GM-CSF**	Adhesion to BEC	Decreased	Not altered
	TEM ^9^ in the presence of rhGM-CSF ^10^	Increased	Not altered
	TEM effect of shCSF2 ^11^	Decreased	Increased
	TEM MBM + rhGM-CSF	Not altered	Not altered
	TEM BEC + rhGM-CSF	Increased	Increased
**in-vivo function of GM-CSF**	Tumorigenesis	Increased	Decreased
	Brain metastasis	Increased	Decreased

Summary of the differential responses of brain metastatic melanoma cells YDFR.CB3 and DP.CB2 to GM-CSF-mediated activation. ^1^ MBM: melanoma brain metastasis, ^2^ NS: not significant, ^3^ MG-CM: microglia-conditioned medium, ^4^ BEC-CM: brain endothelial cell-conditioned medium, ^5^ HA-CM: human astrocyte-conditioned medium, ^6^ IL-1α: interleukin-1α, ^7^ TNF-α: tumor necrosis factor-α, ^8^ NA: not applicable, ^9^ TEM: transendothelial migration, ^10^ rhGM-CSF: recombinant human granulocyte-macrophage colony-stimulating factor, ^11^ shCSF2: shRNA targeting GM-CSF.
